# THE RESPITE FOR ALL PROGRAM DECREASES STIGMA AND CAREGIVERS' ANXIETY WHILE INCREASING THEIR CONFIDENCE

**DOI:** 10.1093/geroni/igac059.190

**Published:** 2022-12-20

**Authors:** Steven Sabat, Abigail Howell, Daphne Johnston

**Affiliations:** Georgetown University, Washington, District of Columbia, United States; Georgetown University, Washington, District of Columbia, United States; First United Methodist Church, Montgomery, Alabama, United States

## Abstract

Feelings of burden and stigma are associated with social isolation (Adelman, et al., 2014) which is considered a public health concern (Brodaty & Donkin, 2009; Tatangelo, et al., 2018a). The stigma associated with a dementia diagnosis, care partners’ burden, and diminishing financial resources have been found to be significant contributors to increased social isolation (Lee, et al., 2021; Sun, et al., 2021; Hung, et al., 2021). Care partners report significant anxiety in connection with behavioral manifestations of dementia attracting negative attention (McHugh, et al, 2012; Sanders, et al, 2008) or judged by the general public (Lee, et al., 2021), and those with high levels of grief commonly report experiencing social isolation (Sanders, et al, 2008). The Respite For All program for people living with dementia has led to decreases in stigma and caregivers’ anxiety while increasing their confidence. We will discuss the nature of this program and present supporting data.

